# Effect of Combination Therapy of Canagliflozin Added to Teneligliptin Monotherapy in Japanese Subjects with Type 2 Diabetes Mellitus: A Retrospective Study

**DOI:** 10.1155/2020/4861681

**Published:** 2020-04-02

**Authors:** Yoshiro Fushimi, Atsushi Obata, Junpei Sanada, Yuichiro Iwamoto, Akiko Mashiko, Megumi Horiya, Akiko Mizoguchi-Tomita, Momoyo Nishioka, Yuki Kan, Tomoe Kinoshita, Seizo Okauchi, Hidenori Hirukawa, Kenji Kohara, Fuminori Tatsumi, Masashi Shimoda, Shuhei Nakanishi, Tomoatsu Mune, Kohei Kaku, Hideaki Kaneto

**Affiliations:** Department of Diabetes, Endocrinology and Metabolism, Kawasaki Medical School, Japan

## Abstract

Recently, dipeptidyl peptidase-4 (DPP-4) inhibitors and sodium-glucose cotransporter 2 (SGLT2) inhibitors have been very often used in subjects with type 2 diabetes mellitus (T2DM). In addition, combination drugs of both inhibitors have attracted much attention in aspects of its cost-effectiveness and improvement of patients' adherence. However, it is still poorly understood which factors are related to the efficacy of SGLT2 inhibitors as add-on therapy to DPP-4 inhibitors. Therefore, we aimed to elucidate in which type of individuals and/or under which conditions canagliflozin as add-on therapy to teneligliptin could exert more beneficial effects on glycemic control and/or renal protection. We retrospectively analyzed 56 Japanese subjects with T2DM in the real-world clinical practice. Three months after starting the combination therapy, the change of HbA1c (*Δ*HbA1c) was strongly related to HbA1c levels at baseline. As expected, serum glucagon level was increased after starting the combination therapy. Interestingly, however, the change of glucagon levels (*Δ*glucagon) was not related to HbA1c levels at baseline, *Δ*HbA1c, and other parameters, which indicated that the increase of glucagon did not clinically affect the effectiveness of combination therapy. In addition, the change of urinary albumin excretion (*Δ*UAE) was negatively correlated with systolic blood pressure and HbA1c levels at baseline and positively correlated with the change of systolic blood pressure (*Δ*sBP) in univariate analysis. Furthermore, in multivariate analysis, only *Δ*sBP was the independent factor associated with *Δ*UAE. Taken together, canagliflozin as add-on therapy to teneligliptin improves glycemic control in a *Δ*glucagon-independent manner and reduces UAE in a *Δ*sBP-dependent manner in Japanese subjects with T2DM.

## 1. Introduction

Recently, DPP-4 inhibitors are commonly used in various individuals with type 2 diabetes mellitus (T2DM) from the young to the elderly because of their good glucose-lowering effect and safety including low risk of hypoglycaemia. DPP-4 inhibitors promote insulin secretion and suppress glucagon secretion by inhibiting the degradation of glucagon-like peptide-1 [1]. SGLT2 inhibitors, which emerged in clinical practice after DPP-4 inhibitors, improve glycemic control by reducing filtered renal glucose reabsorption from the proximal tubule and promoting excretion of excess glucose, which is a totally different mechanism compared with DPP-4 inhibitors [2]. SGLT2 inhibitors are promising drugs in view of reduction of glucose toxicity, body weight, and insulin resistance [3, 4]. In addition, large clinical trials have shown that SGLT2 inhibitors improved 3-point major adverse cardiovascular events (MACE) and renal outcomes [5–7], which were not observed with DPP-4 inhibitors. Since DPP-4 inhibitors and SGLT2 inhibitors have such different mechanisms of action and the risk of hypoglycaemia with both medications is pretty low, it seems that the combination therapy of both inhibitors is very promising. In fact, large clinical trials have shown the efficacy and safety of combination therapy in Japanese individuals with type 2 diabetes [8, 9]. In addition, very recently, combination drugs of both inhibitors became available in clinical practice and have drawn much attention in aspects of its cost-effectiveness and enhancement of patients' adherence. However, it is still poorly understood which factors and/or features of individuals mainly contribute to the glucose-lowering and/or renal protection effects of such combination therapy in real-world clinical practice. Therefore, we aimed to address the answer of these questions in this study.

## 2. Methods

### 2.1. Study Design

The present study was retrospectively carried out with outpatients in the Division of Diabetes, Endocrinology and Metabolism in Kawasaki Medical School from February to December in 2018. The subjects who had been taking teneligliptin (20 mg/day) for longer than 6 months without any other medication changes were included.

The participants were those who fulfilled the following criteria: (i) without severe renal dysfunction (eGFR < 30 mL/min/1.73 m^2^); (ii) without any insulin and/or GLP-1RA usage; (iii) without infectious disease, malignancy, or diabetic coma; and (iv) not using steroid and/or immunosuppressant medications. A total of 91 participants (48 males, 43 females) were enrolled in the present study, and canagliflozin (100 mg/day) was added on to the subjects by each doctor's judgement. As a result, 56 out of 91 participants received canagliflozin (100 mg/day) and the remaining 35 participants continued teneligliptin. After starting canagliflozin add-on therapy for 3 months, blood and urinary samples after overnight fasting were obtained from all patients for measuring the multiple metabolic parameters. Serum glucagon levels were measured by SRL (Glucagon ELISA, Cosmic, Mercodia AB, Sylveniusgatan 8A, 75450 Uppsala, Sweden). The study protocol was approved by the institutional review board of Kawasaki Medical School (No. 3236), and the study was carried out in accordance with the Declaration of Helsinki, and we provided public information on the study via the Internet, instead of obtaining informed consent from each patient. We carried out data collection for variables such as type of medication as well as biochemical data.

### 2.2. Evaluation of Body Composition Parameters

Body composition variables were measured by a bioelectrical impedance body composition analyzer InBody 770 (InBody Japan Inc., Japan). Measurements were performed with the participants barefoot and in minimal clothing without any metallic materials. Body weight, total body water, intracellular and extracellular water, body fat mass, percent body of fat, soft lean mass, and skeletal muscle mass were measured before and after canagliflozin add-on therapy.

### 2.3. Statistical Analysis

All data were analyzed using JMP version 11.0 and are presented as mean ± standard deviation (SD) or number (percentage). Changes in parameters from the baseline values in the same patient were evaluated using Wilcoxon singed-rank test. Baseline parameters and changes of various parameters of the teneligliptin continued group and canagliflozin add-on group were analyzed using Wilcoxon rank sum test. To examine which factors are associated with change (*Δ*) in HbA1c, glucagon, and UAE, we carried out Spearman's rank correlation coefficient test. Furthermore, to examine which factors independently determine *Δ*UAE after 12 weeks of canagliflozin add-on therapy, we carried out multiple regression analysis. *p* value < 0.05 was considered to be statistically significant.

## 3. Results

### 3.1. Clinical Characteristics of the Study Subjects

As shown in Table 1, clinical characteristics of the study subjects were as follows: in the teneligliptin continued group: age, 68.3 ± 11.4 years old; number (male/female), 35 (21/14); duration of diabetes, 17.8 ± 12.9 years; body weight, 63.1 ± 16.4 kg; BMI, 24.3 ± 5.4 kg/m^2^; HbA1c, 6.9 ± 0.6%; systolic blood pressure, 125 ± 13 mmHg; diastolic blood pressure, 71 ± 10 mmHg; triglyceride, 114 ± 51 mg/dL; LDL cholesterol, 96.6 ± 22.8 mg/dL; HDL cholesterol, 54.1 ± 14.1 mg/dL; and uric acid, 5.6 ± 1.3 mg/dL. The prevalence rates of subjects with diabetic microangiopathy were as follows: peripheral neuropathy 40%, retinopathy 26%, and nephropathy stage 1 60%, stage 2 20%, and stage 3 20%, respectively. The prevalence of subjects with history of myocardial infarction and/or stroke was 34%. The prevalence of concomitant medication was as follows: DPP-4 inhibitor 100% (teneligliptin 20 mg/day), biguanide 46%, sulfonylurea or glinide 37%, thiazolidine 34%, and *α*-glucosidase inhibitor 29%. Insulin and/or GLP-1 receptor agonist users were not included in this study.

On the other hand, in the canagliflozin add-on group, the clinical characteristics of the study subjects were as follows: age, 61.6 ± 13.5 years old; number (male/female), 56 (27/29); duration of diabetes, 11.3 ± 8.2 years; body weight, 72.3 ± 20.2 kg; BMI, 28.6 ± 6.7 kg/m^2^; HbA1c, 7.6 ± 1.5%; systolic blood pressure, 133 ± 20 mmHg; diastolic blood pressure, 80 ± 14 mmHg; triglyceride, 157 ± 143 mg/dL; LDL cholesterol, 103.4 ± 32.9 mg/dL; HDL cholesterol, 52.2 ± 15.0 mg/dL; and uric acid, 5.4 ± 1.5 mg/dL. The prevalence rates of subjects with diabetic microangiopathy were as follows: peripheral neuropathy 14%, retinopathy 12%, and nephropathy stage 1 73%, stage 2 18%, and stage 3 9%, respectively. The prevalence of subjects with history of myocardial infarction and/or stroke was 9%. The prevalence of concomitant medication was as follows: DPP-4 inhibitor 100% (teneligliptin 20 mg/day), biguanide 46%, sulfonylurea or glinide 19%, thiazolidine 20%, and *α*-glucosidase inhibitor 2%. As you can see in Table 1, the teneligliptin continued group presented significantly older age, longer duration of diabetes, and lower body weight, FPG, HbA1c, blood pressure, ALT, *γ*GTP, and eGFR at baseline compared with the canagliflozin add-on group. No severe adverse events were observed in this study.

### 3.2. Effects of Combination Therapy of Canagliflozin Added to Teneligliptin Monotherapy on Various Metabolic Parameters

Three months after starting canagliflozin as add-on therapy to teneligliptin, body weight, BMI, HbA1c, fasting blood glucose levels, both systolic and diastolic blood pressures, urinary albumin excretion (UAE), urinary acid, and transaminase were all significantly reduced compared to those before such combination therapy (Table 1). Body weight was decreased by approximately 1.3 kg (72.3 ± 20.2 kg to 71.0 ± 20.2 kg, *p* < 0.0001), and HbA1c levels were decreased by approximately 0.7% (7.6 ± 1.5% to 6.9 ± 0.7%, *p* < 0.0001). In body composition, fat mass was significantly decreased (27.7 ± 14.2 kg to 26.3 ± 14.2 kg, *p* < 0.0001, respectively) accompanied with slight skeletal muscle mass reduction (decrease by approximately 0.2 kg), which was quite little compared to the reduction of fat mass (decrease by approximately 1.4 kg). Both systolic and diastolic blood pressures were decreased after starting canagliflozin as add-on therapy to teneligliptin (133 ± 20 mmHg to 129 ± 17 mmHg(*p* = 0.0041) and 80 ± 14 mmHg to 78 ± 12 mmHg (*p* = 0.032), respectively). Urinary albumin excretion was also significantly reduced after such combination therapy (101 ± 228 mg/g. Cre to 72 ± 172 mg/g. Cre, *p* = 0.020). In contrast, ketone body level was significantly increased (114 ± 117 *μ*mol/L to 141 ± 154 *μ*mol/L, *p* = 0.024). Furthermore, interestingly, the serum glucagon level was significantly increased after starting the combination therapy (149 ± 40 pg/mL to 163 ± 53 pg/mL, *p* = 0.0010).

On the other hand, no variable changes were observed in the teneligliptin continued group between baseline and after three months. We next compared the changes of various parameters between baseline and 3 months later. As shown in Table 2, the decreased changes of body weight, BMI, FPG, and HbA1c were all significantly larger in the canagliflozin add-on group compared with the teneligliptin continued group. In addition, the decreased changes of *γ*GTP, eGFR, and uric acid were also significantly larger accompanied with the significant increased change of Cre in the canagliflozin add-on group.

### 3.3. Search for Possible Factors Which Are Strongly Associated with Glucose-Lowering Effect and the Increase of Serum Glucagon Levels after Combination Therapy

We next investigated which factors were strongly associated with the glucose-lowering effect of canagliflozin as add-on therapy to teneligliptin. First, since HbA1c levels were significantly reduced after starting the combination therapy (Table 1), we evaluated which factors were closely associated with *Δ*HbA1c in univariate analysis. However, only HbA1c levels at baseline were strongly associated with *Δ*HbA1c (data not shown). Next, since serum glucagon levels were significantly elevated after the combination therapy (Table 1), we analyzed which factor levels at baseline were strongly associated with *Δ*glucagon. As shown in Table 3, however, *Δ*glucagon was not significantly related to HbA1c, IRI, and FPG at baseline in the univariate analysis. In addition, we examined the possible association of *Δ*glucagon with the change of various parameters such as *Δ*HbA1c, *Δ*IRI, and *Δ*FPG. However, there were no factors which were strongly associated with *Δ*glucagon, indicating that the elevation of glucagon levels did not affect the glucose-lowering effect in such combination therapy.

### 3.4. Search for Possible Factors Which Are Strongly Associated with the Reduction of Urinary Albumin Excretion (UAE) after Combination Therapy

Since UAE was significantly reduced after starting canagliflozin as add-on therapy to teneligliptin (Table 1), we next investigated which factors were closely associated with the reduction of UAE (*Δ*UAE). First, in the univariate analysis, *Δ*UAE was significantly related to sBP, HbA1c levels at baseline, and *Δ*sBP (Table 4). Next, to elucidate the independent factors determining the efficacy of combination therapy, we performed multivariate analysis. To avoid the possible influence of age or gender, we included age and gender as independent variables as well and thus, we performed multivariate analysis using age, gender, sBP, and HbA1c levels at baseline and *Δ*sBP as explanatory variables and *Δ*UAE as an objective variable. As shown in Table 5, even after adjustment with age and gender, *Δ*sBP was an independent factor contributing to the reduction of UAE after combination therapy.

## 4. Discussion

In this study, we showed that canagliflozin as add-on therapy to teneligliptin exerted beneficial effects on metabolic parameters such as body weight, HbA1c, and uric acid without any severe adverse events (Table 1), which were compatible with the previous reports about the effectiveness of SGLT2 inhibitors [10–12]. In addition, in the univariate analysis, HbA1c levels at baseline were strongly associated with *Δ*HbA1c (data not shown). Unexpectedly, lipid profile showed no significant improvement in this study (Table 1). As the lipid profile at baseline was relatively good in this study, it might have been hard to observe further improvement. It is also possible that as this study was retrospective, we could not educate exercise and/or diet adequately.

In this study, it is hard to directly compare the teneligliptin continued group and canagliflozin add-on group as participants' baseline characteristics were totally different. As canagliflozin was added on by each doctor's judgement, teneligliptin might be continued for patients whose HbA1c was relatively good and body weight was relatively low. It is true that there are some data defects such as fat mass, skeletal muscle mass, IRI, glucagon, IRI/glucagon ratio, ketone body, and urinary albumin excretion as this is a retrospective study. However, there were no other parameter changes after 3 months; we assume that there would be no change in parameters which were not measured in this study. On the contrary, we also explored the changes of various parameters between baseline and 3 months later (Table 2). As expected, the difference of changes such as body weight, BMI, FPG, and HbA1c was significantly larger in the canagliflozin add-on group. In addition, the difference of changes of Cre and eGFR became significant, which was not significantly different in each group comparison (Table 1). It is known that an initial eGFR drop is observed when SGLT2 inhibitors are used. This might partially explain the significant changes of Cre and eGFR in our study. However, unexpectedly, no significant difference in the changes of systolic and diastolic blood pressure was observed in our study. This might derive from some difference of the patients' background of each group or limited small sample size.

In general, it is said that SGLT2 inhibitors decrease serum insulin levels and increase serum glucagon levels, which lead to decrease of the insulin/glucagon ratio. However, in this study, IRI was not reduced after 3 months of combination therapy and there was no change in the IRI/glucagon ratio. There are some possible hypotheses that IRI was not decreased in this study. First, there were some patients who were recovering from glucose toxicity after adding canagliflozin. In these subjects, IRI levels were increased drastically. As sample size was rather small in this study, these subjects might have influenced the total IRI average. Second, as this study is retrospective, the difference of fasting time of each patient might have led to this result.

On the contrary, the serum glucagon level was significantly elevated after starting canagliflozin as add-on therapy to teneligliptin. Furthermore, the univariate analysis revealed *Δ*glucagon was not significantly associated with HbA1c, IRI, and FPG at baseline and *Δ*HbA1c, *Δ*IRI, and *Δ*FPG. These results suggested that the increase of glucagon had no or little impact on glycemic control. Several previous studies reported that treatment with an SGLT2 inhibitor for 1–12 weeks significantly increased fasting or postprandial plasma glucagon levels in type 2 diabetes patients [13–15]. On the contrary, there are some reports which suggest that canagliflozin does not increase the serum glucagon level during meal tolerance test [8, 16]. In addition, it is also presented that SGLT1 but not SGLT2 is expressed in the murine *α*TC1 cell line and human and murine pancreatic islets and that canagliflozin reduced glucagon secretion from *α*TC1 cells in an SGLT1-dependent manner [17]. On the other hand, it is also reported that SGLT2 but not SGLT1 is expressed in glucagon-secreting alpha cells of human and murine pancreatic islets and dapagliflozin triggers glucagon secretion [18]. From the results of these reports, it is possible that some drug effects exist due to the difference of each drug affinity to SGLT1 and SGLT2. It will need further investigation to elucidate whether there are some drug effects on the changes of glucose metabolism and glucagon secretion. Although it is still controversial, we think it is important that our study showed that serum glucagon change did not affect overall metabolic changes in an adverse manner.

Noda et al. reported that teneligliptin and teneligliptin/canagliflozin combination therapy improved blood glucose levels after meal tolerance test (MTT) in 12 participants in a hospitalized setting for 14 days [16]. Interestingly, absolute values of glucagon and active GLP-1 were elevated after starting canagliflozin combination therapy, while *Δ*glucagon was significantly reduced and *Δ*active GLP-1 was increased during MTT compared to teneligliptin monotherapy. This study presented that canagliflozin actually increased glucagon levels, which was similar to our study. However, *Δ*glucagon during MTT was significantly reduced, which could not be confirmed in our study as we did not conduct MTT.

Okahata et al. reported that canagliflozin and canagliflozin/teneligliptin combination therapy improved blood glucose levels after MTT in 26 participants in a hospitalized setting for 6 days [19]. This study presented that canagliflozin increased that serum glucagon level during MTT and addition of teneligliptin reversed this increase. They also evaluated the change of the IRI/glucagon ratio during MTT, which resulted in no difference between canagliflozin treatment and canagliflozin/teneligliptin treatment.

These two studies suggest that addition of canagliflozin actually increases the serum glucagon level. Furthermore, the latter study presented no IRI/glucagon ratio difference during MTT between canagliflozin therapy and canagliflozin/teneligliptin combination therapy. On the contrary, these studies are in an inpatient setting, and the intervention term was quite short, and patients' backgrounds such as age and disease duration are totally different from our study.

Kadowaki et al. reported two large randomized control studies regarding the canagliflozin/teneligliptin combination vs. the monotherapy [8, 9]. One was canagliflozin added on to teneligliptin. The other was vice versa. Canagliflozin added on to teneligliptin improved glucose levels during MTT. However, the serum glucagon level was not increased in this study. This result might have derived from the method of glucagon measurements. In their study, glucagon was measured by radioimmunoassay (RIA). It is reported that there is crossreactivity against several proglucagon-derived fragments in RIA for glucagon determination. Some reports have elucidated a large difference in glucagon results in the same sample between RIA and sandwich ELISA [20, 21]. This might partially explain the difference of the glucagon result from our study. Anyway, all studies including our study presented that canagliflozin add-on therapy to teneligliptin improved various metabolic parameters.

Next, in this study, we presented that *Δ*UAE was significantly related to sBP and HbA1c levels at baseline and *Δ*sBP. Furthermore, multivariate analysis showed that *Δ*sBP was an independent factor contributing to the reduction of UAE after combination therapy. Several large clinical trials reported that SGLT2 inhibitors such as empagliflozin, canagliflozin, and dapagliflozin reduced the incidence of end-stage kidney disease, doubling of the serum creatinine levels from baseline, or death from renal or cardiovascular disease [5–7]. Although there was no change in eGFR after starting combination therapy in this study, such *Δ*sBP-dependent reduction of UAE might lead to a renal protective effect of canagliflozin add-on therapy in the long term.

Needless to say, there are limitations in this study. First, since this study was retrospective and our sample size was small, a large-scale and randomized prospective study would be necessary to achieve higher levels of evidence. Second, since most of the subjects in this study had mild to moderate renal dysfunction, the data obtained in this study is not necessarily true for subjects with severe renal dysfunction. Third, since these study subjects were middle aged and relatively obese, the data obtained in this study is not necessarily true for relatively aged and lean subjects. In addition, it is known that dual-energy X-ray absorptiometry is a gold standard method for the evaluation of body composition, but in this study, we used bioelectric impedance assessments to evaluate body composition because of their simplicity and portability.

Taken together, treatment with canagliflozin in addition to teneligliptin showed beneficial effects on various metabolic parameters and significantly reduced urinary albumin excretion. Furthermore, multivariate analyses showed that the reduction of urinary albumin excretion was mainly determined by the decrease of blood pressure. In addition, the change of serum glucagon levels was not related to any metabolic parameters, suggesting that the increase of glucagon levels after adding canagliflozin did not mainly, if any, contribute to the aggravation of overall metabolism. In conclusion, treatment with canagliflozin in addition to teneligliptin decreases urinary albumin excretion, at least in part, through the reduction of blood pressure and improves a variety of metabolic parameters in a glucagon-independent manner in Japanese subjects with type 2 diabetes mellitus.

## Figures and Tables

**Table 1:** Efficacy of canagliflozin add-on therapy on various metabolic parameters.

Parameters	Baseline	12 weeks	*p*	Baseline	12 weeks	*p*
Group	Teneligliptin continued group			Canagliflozin add-on group		
Age (years)	68.3 ± 11.4	—	—	61.6 ± 13.5^∗^	—	—
Gender (M/F)	21/14	—	—	27/29^∗^	—	—
DM duration (years)	17.8 ± 12.9	—	—	11.3 ± 8.2^∗^	—	—
Body weight (kg)	63.1 ± 16.4	63.8 ± 16.6	n.s.	72.3 ± 20.2^∗^	71.0 ± 20.2	<0.0001
BMI (kg/m^2^)	24.3 ± 5.4	24.5 ± 5.4	n.s.	28.6 ± 6.7^∗^	27.7 ± 6.7	<0.0001
Fat mass (kg)	—	—	—	27.7 ± 14.2	26.3 ± 14.2	<0.0001
Visceral fat area (cm^2^)	—	—	—	137 ± 62	129 ± 64	<0.0001
Skeletal muscle mass (kg)	—	—	—	25.1 ± 7.5	24.9 ± 7.5	0.047
FPG (mg/dL)	138 ± 22	134 ± 27	n.s.	166 ± 51^∗^	139 ± 27	<0.0001
HbA1c (%)	6.9 ± 0.6	6.9 ± 0.6	n.s.	7.6 ± 1.5^∗^	6.9 ± 0.7	<0.0001
IRI (*μ*IU/mL)	—	—	—	12.3 ± 9.7	16.4 ± 20.2	n.s.
Glucagon (pg/mL)	—	—	—	149 ± 40	163 ± 53	0.0010
IRI/glucagon	—	—	—	0.09 ± 0.07	0.09 ± 0.09	n.s.
Systolic BP (mmHg)	125 ± 13	124 ± 13	n.s.	133 ± 20^∗^	129 ± 17	0.0041
Diastolic BP (mmHg)	71 ± 10	70 ± 10	n.s.	80 ± 14^∗^	78 ± 12	0.032
AST (U/L)	24.2 ± 9.5	22.4 ± 8.1	n.s.	28.4 ± 18.0	26.3 ± 11.2	n.s.
ALT (U/L)	20.7 ± 12.1	19.4 ± 12.4	n.s.	27.9 ± 12.7^∗^	26.4 ± 9.0	0.031
*γ*GTP (U/L)	30.3 ± 35.2	29.4 ± 30.1	n.s.	46.8 ± 51.6^∗^	39.3 ± 23.9	0.0005
Cre (mg/dL)	0.8 ± 0.3	0.8 ± 0.3	n.s.	0.7 ± 0.2	0.7 ± 0.2	n.s.
BUN (mg/dL)	17.9 ± 5.3	17.2 ± 5.7	n.s.	15.2 ± 4.5^∗^	17.5 ± 4.5	<0.0001
eGFR (mL/min/1.73 m^2^)	69.7 ± 18.7	72.9 ± 21.0	n.s.	81.2 ± 21.7^∗^	79.7 ± 23.9	n.s.
Triglyceride (mg/dL)	114 ± 51	106 ± 44	n.s.	157 ± 143	163 ± 186	n.s.
HDL cholesterol (mg/dL)	54.1 ± 14.1	54.1 ± 15.0	n.s.	52.2 ± 15.0	50.2 ± 12.0	n.s.
LDL cholesterol (mg/dL)	96.6 ± 22.8	92.4 ± 21.8	n.s.	103.4 ± 32.9	103.5 ± 29.2	n.s.
Ketone body (*μ*mol/L)	—	—	—	114 ± 117	141 ± 154	0.024
Uric acid (mg/dL)	5.6 ± 1.3	5.4 ± 1.4	n.s.	5.4 ± 1.5	4.9 ± 0.8	<0.0001
Urinary albumin excretion (mg/g. Cre)	148 ± 285	—	—	101 ± 228	72 ± 172	0.020
Urinary glucose excretion (mg/g. Cre)	—	—	—	71 ± 478	2672 ± 2126	<0.0001

^∗^Teneligliptin continued group *vs.* canagliflozin add-on group at baseline (Wilcoxon rank sum test), *p* < 0.05.

**Table 2:** Changes of various parameters between baseline and 3 months later.

Parameters	Teneligliptin continued group	Canagliflozin add-on group	*p*
*Δ*Body weight (kg)	+0.7 ± 1.5	−1.3 ± 1.9	<0.0001
*Δ*BMI (kg/m^2^)	+0.2 ± 0.6	-0.9 ± 0.7	<0.0001
*Δ*FPG (mg/dL)	−4.3 ± 22.1	−27.3 ± 40.3	0.0003
*Δ*HbA1c (%)	−0.02 ± 0.4	−0.7 ± 0.8	<0.0001
*Δ*Systolic BP (mmHg)	−0.4 ± 12.2	−4.6 ± 10.5	n.s.
*Δ*Diastolic BP (mmHg)	−0.2 ± 9.7	−2.6 ± 9.4	n.s.
*Δ*AST (U/L)	−1.8 ± 6.3	−2.1 ± 9.1	n.s.
*Δ*ALT (U/L)	−1.3 ± 6.4	−1.5 ± 11.6	n.s.
*Δγ*GTP (U/L)	−0.9 ± 9.8	−7.5 ± 22.5	0.03
*Δ*Cre (mg/dL)	−0.02 ± 0.07	+0.02 ± 0.07	0.01
*Δ*BUN (mg/dL)	−0.7 ± 3.9	+2.3 ± 3.3	0.001
*Δ*eGFR (mL/min/1.73 m^2^)	+3.2 ± 8.6	−1.5 ± 8.8	0.04
*Δ*Triglyceride (mg/dL)	−8.0 ± 40.7	+6.2 ± 70.2	n.s.
*Δ*HDL cholesterol (mg/dL)	−0.02 ± 4.8	−2.1 ± 10.6	n.s.
*Δ*LDL cholesterol (mg/dL)	−4.2 ± 13.6	+0.1 ± 19.6	n.s.
*Δ*Uric acid (mg/dL)	−0.2 ± 0.6	−0.6 ± 0.7	0.02

**Table 3:** Various parameters associated with *Δ*glucagon in univariate analyses.

Comparison with baseline value	*ρ*	*p*
Age (years)	-0.0088	n.s.
Gender	0.0426	n.s.
Baseline FPG (mg/dL)	-0.0936	n.s.
Baseline HbA1c (%)	-0.1502	n.s.
Baseline IRI (*μ*IU/mL)	0.1216	n.s.

Comparison with change of each value	*ρ*	*p*
Age (years)	-0.0088	n.s.
Gender	0.0426	n.s.
*Δ*FPG (mg/dL)	0.0582	n.s.
*Δ*HbA1c (%)	0.1366	n.s.
*Δ*IRI (*μ*IU/mL)	-0.1756	n.s.

**Table 4:** Various parameters associated with *Δ*urinary albumin excretion (*Δ*UAE) in univariate analyses.

Comparison with baseline value	*ρ*	*p*
Age (years)	-0.2571	n.s.
Gender	-0.1770	n.s.
Baseline systolic blood pressure (mmHg)	-0.3006	0.038
Baseline urinary Na^+^ excretion (mEq/L)	0.1419	n.s.
Baseline HbA1c (%)	-0.3073	0.027

Comparison with change of each value	*ρ*	*p*
Age (years)	-0.2571	n.s.
Gender	-0.1770	n.s.
*Δ*Systolic blood pressure (mmHg)	0.4660	0.0008
*Δ*Urinary Na^+^ excretion (mEq/L)	-0.0428	n.s.
*Δ*HbA1c (%)	0.0659	n.s.

**Table 5:** Determinant factors associated with *Δ*urinary albumin excretion (*Δ*UAE) in multivariate analyses.

Explanatory variables	*β*	*p*
Age (years)	0.0726	n.s.
Gender	0.0502	n.s.
Baseline systolic blood pressure (mmHg)	-0.0222	n.s.
Baseline HbA1c (%)	-0.166	n.s.
*Δ*Systolic blood pressure	0.417	0.01

## Data Availability

The datasets used and/or analyzed during the current study are available from the corresponding author on reasonable request.
